# Development and validation of the ASKFV-SE tool to measure children's self-efficacy for requesting fruits and vegetables

**DOI:** 10.1017/jns.2022.59

**Published:** 2023-01-06

**Authors:** Sarah Amin, Sabrina Hafner, Jade McNamara, Joanna Raymond, Kate Balestracci, Amanda Missimer, Jacquelyn Potvin, Geoffrey Greene

**Affiliations:** 1 Department of Nutrition and Food Sciences, College of Health Sciences, University of Rhode Island, Kingston RI, 02881, USA; 2 School of Food and Agriculture, University of Maine, Orono, ME 04469, USA

**Keywords:** Children, Fruits, Self-efficacy, Vegetables

## Abstract

The aim of the present study was to develop the ASKFV-SE tool to measure self-efficacy (SE) towards requesting fruits and vegetables (FV) in the home and school environment with school-age children (grades 4–5) from urban, ethnically diverse, low-income households. Cognitive interviews reduced the tool from eleven items to seven. The 7-item questionnaire was tested with 444 children. The items loaded on two factors: home SE (four items) and school SE (two items) with one item was excluded (<0⋅40). The reduced 6-item, 2-factor structure was the best fit for the data (*χ*^2^ = 45⋅09; df = 9; CFI = 0⋅835; RMSEA = 0⋅147). Confirmatory factory analysis revealed that the 4-item home SE had high reliability (*α* = 0⋅73) and marginally acceptable reliability for the 2-item school SE (*α* = 0⋅53). The pre-COVID intra-class correlation coefficient (ICC) was 0⋅584 (*P* < 0⋅001; fair; *n* = 57) compared to 0⋅736 during-COVID (*P* < 0⋅001; good; *n* 50). The ASKFV-SE tool measures children's SE for asking for FVs with strong psychometric properties and low participant burden.

## Introduction

Healthy dietary patterns include consuming adequate amounts of fruits and vegetables (FV). Low consumption of FV in childhood is associated with an increased risk of chronic diseases in adulthood^(1)^. Children in the United States aged 5–13 years consume less than half the recommended amount of FV, and those from low socioeconomic status (SES) families consume less FV than children of higher-SES families^(1)^.

Between 5 and 13 years is a critical time to establish healthy dietary behaviours^(2)^. During these years, children transition from food provided by primary caregivers to more autonomy to choose, thereby establishing lifelong eating habits^(2)^. According to previous research, modifications in accessibility, awareness and preference have a significant impact in reported FV consumption^(3,4)^.

Self-efficacy (SE), a person's confidence in their ability to perform a particular behaviour, is one of the most widely accepted determinants of dietary behaviour change^(5–7)^. However, diet-related SE requires perceived control over the food environment, which is challenging for preadolescents. A more appropriate intervention target for these children is asking SE, defined as confidence in asking adults for a specific type of food. Children's SE for consuming FV was first assessed in 1996 and revised in 2000 to include a dimension measuring SE in asking and shopping for FV. In 2011, the tool was streamlined to 11 items which was found to be reliable and sensitive to change in 9–11-year-old children experiencing low-income^(8–10)^. However, asking and shopping were combined in the same scale which is problematic because the behaviours differ and most preadolescent children do not shop for produce. In addition, the instrument was not psychometrically validated and did not assess FV asking SE at school, which is important given that children from low-SES households consume the majority of their meals at school^(11)^.

Existing tools do not measure the construct of asking for FV, especially among preadolescent children from low-income, ethnically diverse households. Therefore, the need to establish a reliable assessment tool for this population that is disproportionately impacted by low FV intake and risk of chronic disease is critical for supporting autonomy for food choices. The purpose of the present study was to develop an assessment tool, ASKFV-SE, measuring elementary-aged children's SE in asking for FV in the school and home environment.

## Methods

The ASKFV-SE tool was adapted from an 11-item, instrument assessing asking and shopping SE (Cronbach's *α* = 0⋅80), which had been tested among 9- to 11-year-old children from low-income households^(8)^. The original 11-item instrument was revised by the research team with the following changes: five items were removed due to not being relevant to asking SE at home or age appropriateness (i.e., grocery shopping) and four items about asking SE at school were added. The ASKFV-SE tool initially went through three rounds of cognitive interviews (CIs) with 5th graders from a low-SES and ethnically diverse school (demographics for all study phases are described in the results), with refinement of the tool after each round. The classroom teacher selected students to participate in the one-on-one CI with a research team member to ensure tool clarity, comprehension, and suitability. The CIs were not recorded, but extensive notes were taken.

Next, survey field-testing with 4th and 5th grade students from low-SES and ethnically diverse schools was conducted. Data were stratified by grade, then randomly split with 50 % for exploratory factor analysis (EFA; *n* 222) and 50 % for confirmatory factor analyses (CFA; *n* 222). For psychometric validation, parameters (MAP test, *χ*^2^, CFI, RMSEA) are listed with loading values of items dropped/loaded on each scale along with the coefficient *α* of the scale.

Lastly, test–retest reliability was conducted with two cohorts of 4th and 5th graders, both from low-SES, ethnically diverse schools. Before the pandemic, the survey was administered in person on paper to students (January–February 2020, *n* 57). During the pandemic, the survey was administered virtually to students (January–February 2021, *n* 50). In both instances, the survey was taken twice, with 1 week between administrations. This study was conducted according to guidelines in the Declaration of Helsinki. All procedures involving human subjects were approved by the University of Rhode Island Institutional (URI) Board (#422286-9). The ASKFV-SE data were collected as part of a URI Supplemental Nutrition Assistance Program Education (SNAP-Ed) direct education program. Verbal consent was witnessed and formally recorded for all participating youth.

## Results

The revised 10-item instrument underwent CI with seventeen 5th grade students (*n* 10 male, *n* 7 female, 95 % free or reduced-price lunch eligible (FRPL), 83 % Hispanic and 8 % Black). Three items that confused students were removed. Lastly, ‘fruit’ and ‘vegetable’ were underlined in two places to help students focus on the subject of the statement. The questionnaire used an anchored, Likert-type, response scale from the original instrument, ‘I disagree very much’, ‘I disagree a little’, ‘I am not sure’, ‘I agree a little’ and ‘I agree very much’.

After completion of CI, a different sample of 4th and 5th graders (*n* 444, 88⋅3 % FRPL, 66⋅1 % Hispanic, 16⋅2 % Black) completed the 7-item SE instrument (five home environment questions, two school environment questions). The sample of 444 was randomly split for EFA and CFA. EFA using Maximum Likelihood extraction, Oblimin with Kaiser Normalisation rotation, found a two-factor solution (Goodness-of-fit *χ*^2^ = 15⋅1, *P* < 0⋅01; 56 % of variance explained) with two scales, home and school (Table 1). The 4-item home scale had factor loadings 0⋅674–0⋅789. One item from the 7-item scale ‘ask for fruit at dinner’ failed to load adequately and was deleted. The 2-item school scale had factor loadings 0⋅78–0⋅86. Using CFA, the six loaded items loaded onto two components, home (Cronbach's *α* = 0⋅73) and school (Cronbach's *α* = 0⋅53) was the best fit for the data (*χ*^2^ = 45⋅09; df = 9; CFI = 0⋅835; RMSEA = 0⋅147; Table 2).

**Table 1.** Asking fruit and vegetable SE factor loadings on home and school component based on exploratory factor analysis

Item	Component 1 = Home	Component 2 = School
I think I can ask someone in my family to buy my favourite fruit or vegetable	0⋅674	−0⋅196
I think I can ask someone in my family to make my favourite vegetable for dinner	0⋅738	0⋅061
I think I can ask someone in my family to serve my favourite fruit at dinner	0⋅381	0⋅441
I think I can ask someone in my family to have fruits where I can reach them	0⋅734	0⋅129
I think I can ask someone in my family to have vegetables cut up where I can reach them	0⋅789	−0⋅005
I think I can ask an adult in my school to offer fruits and vegetables I like to eat	0⋅133	0⋅778
I think I can ask an adult at school to change foods offered in my school	−0⋅035	0⋅855

**Table 2.** Asking fruit and vegetable SE factor loadings based on confirmatory factor analysis

Item	Factor loadings
Home Overall (*α* = 0⋅73)	
I think I can ask someone in my family to buy my favourite fruit or vegetable	0⋅60
I think I can ask someone in my family to make my favourite vegetable for dinner	0⋅62
I think I can ask someone in my family to have fruits where I can reach them	0⋅53
I think I can ask someone in my family to have vegetables cut up where I can reach them	0⋅83
School Overall (*α* = 0⋅53)	
I think I can ask an adult in my school to offer fruits and vegetables I like to eat	0⋅69
I think I can ask an adult at school to change foods offered in my school	0⋅53

Lastly, test–retest reliability was conducted with 5th grade students at two different timepoints: pre-pandemic during November 2019 (*n* 57, 79 % FRPL, 38 % Hispanic and 9 % Black) and during-pandemic in January 2021 (*n* 50, 76 % FRPL, 56 % Hispanic and 24 % Black). Overall, the pre-COVID intra-class correlation coefficient (ICC) was 0⋅584 (*P* < 0⋅001; fair) compared to 0⋅736 during-COVID (*P* < 0⋅001; good) (Table 3).

**Table 3.** Test–retest reliability of asking fruit and vegetable SE instrument

Timepoint	Home ICC	School ICC	Overall ICC
Pre-COVID (*n* 57)	0⋅544**	0⋅653**	0⋅584**
During-COVID (*n* 50)	0⋅761**	0⋅295*	0⋅736**

During-COVID, ICCs were collected during distance learning.

ICC, intra-class correlation coefficient.

* *P* < 0⋅05.

** *P* < 0⋅01.

## Discussion

SE in asking for FV is challenging in school-age children. While preadolescents are starting to become more independent, their food choices and behaviours may be different between the home and school environments, particularly among ethnically diverse children experiencing low income. There are limited tools that address both the home and school environments, especially with strong psychometric support. Overall, our findings show the final 6-item questionnaire is a psychometrically valid and reliable tool for assessing FV asking SE in home and school settings^(12)^.

Although the construct of SE has been found to be a key determinant of behaviour change and is critical to many theories, the construct must be operationalised in terms of behaviours and for specific populations. Interventions for adults and adolescents have been found to increase both FV SE and FV consumption^(13)^. However, there is limited research in children, and results have been mixed^(14)^. A 24-question survey measuring perceived SE in 4th graders demonstrated varied outcomes when stratified by both SES and ethnicity, indicating that the tool was not particularly sensitive to these two demographic factors and revisions were required^(15)^. Conversely, a tool that measured SE in children and FV proxy efficacy, or a child's confidence in skills and abilities to get others to act in that individual's interests, found that White children from higher-SES households had more influence on parental FV purchasing and serving of FV at meal times compared to those from lower-SES and more diverse households^(16)^. Another tool evaluating FV, water and physical activity SE found varied, inconsistent and unreliable results based on children's height and body weight^(17)^. A follow-up study utilising an abbreviated version of that tool still found issues and inconsistences in confidence and diversity in dietary intake^(18)^. The mixed results in the literature clearly demonstrate the need for this psychometrically strong ASKFV-SE tool.

The major strengths of this ASKFV-SE tool are its rigorous development and psychometric testing leading to a tool that is developmentally appropriate and accounts for the unique needs of preadolescent children from ethnically diverse, low-income households. This 6-item survey was feasible to administer to students, and administrators reported that the tool required less explanation compared to other surveys. Students completed the survey in approximately 3–5 min and administrators reported it was easy for the students to grasp the content. Importantly, ASKFV-SE encompasses the home and school, which are two critical environments in the socio-ecological model for positively shaping the FV behaviours of children^(19)^. This tool can be administered either on paper or electronically, promoting flexibility for researchers to use in person or virtually.

One unforeseen limitation of the present study is that test–retest reliability was conducted both before and during COVID-19. Due to the environmental context of these two data points, there were differences in the home and school domains, suggesting that environmental context could have impacted reliability. More specifically, test–retest reliability was assessed before COVID in school and during-COVID during distance learning. Interestingly, the school ICCs (0⋅653 pre-COVID *v*. 0⋅295 during-COVID) and home ICCs (0⋅544 pre-COVID *v*. 0⋅761 during-COVID) were markedly different, although acceptable. This unusual circumstance reflects challenges in assessing reliability during a pandemic. While the ICCs varied depending on the environment, they were associated with where students were at the time (home *v*. school) which can also be viewed as a strength for researchers looking to administer the tool in diverse settings. Other limitations include language availability (English only), and generalisability since the ASKFV-SE was developed with urban children from ethnically diverse, low-SES households.

A next step in this research is to assess whether change in SE in asking for FV in children is associated with change in FV consumption behaviour and whether this change varies in the home or school environment. The connection between SE and dietary consumption of FV has been demonstrated in adult studies^(13)^. Little consistent data is available for this outcome in children who are at risk, including objectively measured dietary data^(14)^. Additionally, at this transitional age, cognitive development and social desirability bias can impact decision making. Therefore, conducting qualitative research may help to understand beliefs, attitudes and behaviours around SE in asking for FV in children who are at risk^(20)^.

In conclusion, the ASKFV-SE is a short, easy to administer, psychometrically valid and reliable survey tool. It can be used in multi-level interventions for measuring asking FV SE at home and at school for urban children from ethnically diverse, low-income households. Importantly, children at this age have limited control over their food environments. For interventions that aim to promote children having a role in their home and school food environments, the only effective mechanism is asking for preferred FV. Other instruments do not assess self-efficacy for this construct. Thus, the ASKFV-SE is a contribution to the toolkit for interventionists who are working on programmes to empower children in school and home settings. This work in asking SE could be expanded to target other dietary behaviours such as whole grains and dairy as well as physical activity.
